# Advances of DNA Damage Repair-Related Drugs and Combination With Immunotherapy in Tumor Treatment

**DOI:** 10.3389/fimmu.2022.854730

**Published:** 2022-02-23

**Authors:** Yumin Wang, Meihan Duan, Zhouying Peng, Ruohao Fan, Yuxiang He, Hua Zhang, Wei Xiong, Weihong Jiang

**Affiliations:** ^1^ Department of Otolaryngology Head and Neck Surgery, Xiangya Hospital, Central South University, Changsha, China; ^2^ National Clinical Research Center for Geriatric Disorders, Xiangya Hospital, Changsha, China; ^3^ School of Medicine, Tsinghua University, Beijing, China; ^4^ Department of Oncology, Xiangya Hospital, Central South University, Changsha, China; ^5^ Key Laboratory of Carcinogenesis and Cancer Invasion of the Chinese Ministry of Education, Cancer Research Institute, Central South University, Changsha, China

**Keywords:** DNA damage, DNA repair, immunotherapy, combination, tumor treatment

## Abstract

Cancer therapy has been an important and popular area in cancer research. With medical technology developing, the appearance of various targeted drugs and immunotherapy offer more choices to cancer treatment. With the increase in drug use, people have found more and more cases in which tumors are resistant to DNA damage repair (DDR)-based drugs. Recently, the concept of combination therapy has been brought up in cancer research. It takes advantages of combining two or more therapies with different mechanisms, aiming to benefit from the synergistic effects and finally rescue patients irresponsive to single therapies. Combination therapy has the potential to improve current treatment of refractory and drug-resistant tumors. Among the methods used in combination therapy, DDR is one of the most popular methods. Recent studies have shown that combined application of DDR-related drugs and immunotherapies significantly improve the therapeutic outcomes of malignant tumors, especially solid tumors.

## Introduction

Genome instability is one of the 10 hallmarks of cancer (1). Cells are exposed to various sources of DNA damage in metabolic activities such as reactive oxygen species (ROS). To cope with it, human cells have developed a complex series of DNA repair strategies to protect the integrity and stability of genome (2). However, when the repair fails, the destruction leads to carcinogenesis and genome instability. Studies show that the formation of many malignant tumors is apparently related to the defects in DNA repair (3). On the other hand, the defects may indicate the weakness of cancer cells in DNA repair, which can be targeted in treatment. Therefore, people have developed a great number of drugs targeting DDR to treat cancer, such as cisplatin and Olaparib. These DDR-related drugs greatly improve the survival time and therapeutic outcomes of cancer patients.

Immunotherapy is a newly developed method emerging these years. At present, there have been different approaches for immunotherapy applied in clinical practice. Targeting the immunological changes in cancer, people designed many strategies acting on various kinds of targets (4). Immunotherapy represented by PD-1/PD-L1 inhibitor and chimeric antigen receptor T cell (CAR-T) has shown satisfactory targeting ability and curative effect in cancer therapy, implying its promising prospect in treatment (5). However, both experiments and clinical trials suggest that immunotherapy has very limited effect in solid tumors. Therefore, improving the outcome of immunotherapy in solid tumors has become a critical issue in recent studies.

With the increase in chemotherapy use, people have found more and more cases in which tumors are resistant to DDR-based drugs. As research moves along, the concept of combination therapy has been brought up in cancer studies (6). It takes advantages of combining two or more therapies with different mechanisms, aiming to benefit from the synergistic effects and finally rescue patients irresponsive to single therapies. Combination therapy has the potential to improve current treatment of refractory and drug-resistant tumors. This review summarizes the progress on the association among DNA repair defects, host immune response, and the tumor sensitivity to immunotherapy.

## Applications and Mechanism of DDR-Related Drugs

**Figure 1:**
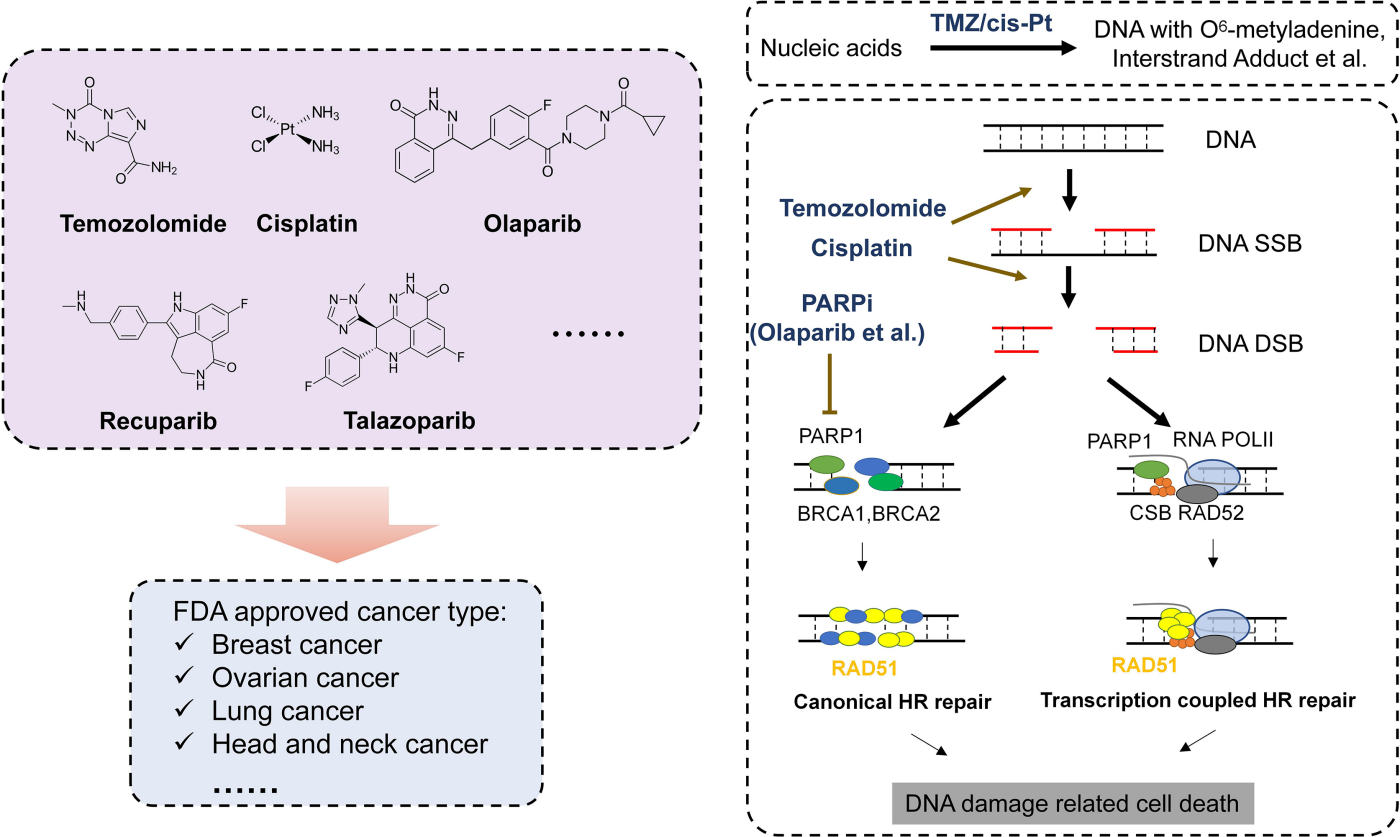
DDR drugs and mechanism of DDR drug in cancer treatment. Left: Represent FDA-approved DNA damage-related drugs. Right: Mechanism of DDR drugs in DNA repair progress.

Complicated mechanisms are necessary to keep genetic code intact from endogenous and exogenous impairment. There are several DDR pathways now that are usually divided into DNA single- and DNA double-strand DNA repair. DNA single-strand repair pathway mainly responds to impaired or mismatched basic groups (e.g., base excision repair, nucleotide excision repair, direct repair, and mismatch repair), while DNA double-strand repair pathway is mainly in charge of DNA double-strand breaks (DSBs) (e.g., homologous recombination, non-homologous end joining, and Fanconi anemia pathway) (7). When DNA repair fails, oncogenes and antioncogenes may obtain somatic mutations, resulting into uncontrolled proliferation and carcinogenesis. It has been found that cancer susceptibility can be caused by several diseases, most of which are associated with genetic defects in certain DNA repair pathways (8). Further studies show that DNA repair defects oftentimes play a vital role in tumor formation and progression. Recently, researchers have analyzed the DNA repair defects in 33 types of cancer. The results show that about one-third of cancers harbor somatic mutations such as BRCA1/2-mutated breast cancer or ovarian cancer and MGMT-methylated glioblastoma, which may give us a hint on the treatment. Both *in vivo* and *in vitro* experiments suggest that chemotherapy-targeting DDR have satisfactory effects on these cancers lacking functional DDR (9) (**Table 1**). Below, we will introduce several representative DDR-related drugs (**Figure 1**).

**Table 1:** DDR-related drugs and combination with immunotherapy in human cancer.

Drug name	Anti-tumor mechanism	Tumor type	Immunotherapy combination
PARPi (Olaparib, etc.)	PARP1, PARP2	Breast cancer; ovarian cancer	Combine with Pembrolizumab in recurrent ovarian cancer
Cisplatin	DNA crosslinker	Sarcoma; carcinoma (lung cancer, head and neck cancer; cervical cancer, etc.)	Combine with Nivolumab and Ipilimumab in NSCLC, combine with PD1 inhibitor in head and neck cancer
Doxorubicin	Anthracycline	Blood disorders; sarcoma; carcinoma (breast, bladder, lung ovarian)	
Etoposide	TOP2	Lung cancer, lymphoma; Ewing’s sarcoma; GBM	Combine with Tezolizumab and carboplatin in small cell lung cancer
Gemcitabine	Pyrimidine antimetabolite	NSCLC; pancreatic cancer; breast cancer; bladder cancer; etc.	
Methotrexate	Antimetabolite	Breast cancer; leukemia; lung cancer; osteosarcoma	
Mitomycin-C	DNA crosslinker	Gastric cancer; pancreatic cancer; bladder cancer	
Pemetrexed	DNA replication	Pleural mesothelioma; non-small cell lung cancer (NSCLC).	
Temozolomide	DNA alkylating agent	Brain cancers; astrocytoma; glioblastoma	Combine with PD-1 inhibitor in glioma
		Olaparib in combination with temozolomide demonstrated substantial clinical activity in relapsed small cell lung cancer	
Camptothecin	TOP1	Gastric cancer; esophageal cancer, cardiac cancer, colon cancer, rectal cancer, primary liver cancer; Acute and chronic myelogenous leukemia, choriocarcinoma, lung cancer, and bladder cancer; Breast cancer	
Carmustine	DNA replication	Glioma, glioblastoma multiforme, medulloblastoma and astrocytoma, multiple myeloma, and lymphoma	
Cyclophosphamide	Alkylating agent	Lymphoma; leukemia; brain cancer	
Epirubicin	Anthracycline	Breast cancer	
Irinotecan	TOP1	Colorectal cancer and small cell carcinoma	
Mitoxantrone	DNA replication	Breast cancer, acute lymphoblastic leukemia, and non-Hodgkin’s lymphoma	
Oxaliplatin	DNA alkylating agent	Colorectal cancer	
Topotecan	TOP1	Ovarian cancer and lung cancer	

### Temozolomide

Temozolomide (TMZ) is one of the widely used chemotherapy drugs targeting DDR. Studies showed that O6 methylguanine (O6 MeG) is the main cytotoxic DNA damage (10). TMZ can methylate DNA, which most often occurs at the N-7 or O-6 positions of guanine residues. Experiments using the cancer cell lines in which high-level methylation or mutation leads to diminished MGMT functions, and transgenic or gene knockout mice experiments all prove that dysfunctional MGMT is one of the main reasons why cells are sensitive to TMZ. In all of the cases and experiments, MGMT deficiency turns cells to be more sensitive to methylating anti-cancer drugs (11). Similarly, when using small-molecule soluble substrates such as O6-benzylguanine and O6-bromothiopheneguanine to inhibit the repair activity of MGMT, the MGMT-tolerant cells turn to be sensitive to TMZ. Moreover, this phenomenon is independent of cancer types. Besides the lethal effect to tumor cells, TMZ also has severe cytotoxicity, mutagenicity, and genotoxicity. The main mechanism of cytotoxicity is mismatch repair (MMR) (12). DNA O6 MeG causes the mismatch of deoxyribonucleotide and thymine, which can be recognized and repaired by MMR complex proteins like MSH2, MSH6, MLH1, and PMS2, resulting in the excision of thymine. In the process, activated EXO1 may produce long nicks (>1,000 bp), which will be repaired later. But during the repair, O6 MeG will match thymine again due to the mismatch feature of lesions. Consequently, a vain, repetitive repair cycle is initiated. Apparently, this is an obstacle to the subsequent DNA replication in S phase (13), and finally, it will cause DNA DSB (14) in MGMT- and MMR+ cancer cells. Although the detailed molecular mechanism has not been fully understood yet, many have verified that DSB produced by O6 MeG processing is lethal by triggering apoptosis. Moreover, it is reported that cells activate apoptosis to detect O6 MeG-T mismatch directly through ataxia-telangiectasia mutated and rad3-related (ATR)/ATR-interacting protein (ATRIP) signal pathway in MTR. This finding also reveals that DDR activation, DSB formation, and the initiation of cell death pathway only happen when DNA replicates twice after DNA damage induction. Therefore, most studies support the model in which DSB formed in the second replication cycle triggers cell apoptosis and finally caused cell death (15). It should be noted that restriction enzyme-induced DSB has high selectivity and efficiency in inducing cell apoptosis, which may explain why TMZ can lead to severe cell apoptosis. Importantly, studies using mutated *Cricetulus griseus* lacking HR pathway show that this cell line is extremely sensitive to O6-methylating agents. The phenomenon strongly supports the model in which HR repairs processed O6 products and generates MeG/MMR intermediate to trigger cell death from another aspect. A study specifically utilizing phosphatase and tensin homolog (PTEN)-lacked primary astrocyte also demonstrates this. The expression of RAD51B, RD51C, and RAD51D is downregulated to weaken HR in the cell line. Since 36% glioma has PTEN mutation, this finding has significant meaning and potential in therapy (16).

### PARP Inhibitors

For now, as the first tumor-targeted drug approved by Food and Drug Administration (FDA), PARPi has attracted much attention from its discovery. Studies have found that various types of human cancer have latent defects in HR pathway, including ovarian cancer, breast cancer, prostate cancer, and pancreas cancer (17). Despite the reasons of their HR dysfunction are not clear, the defect does provide more genome instability for cancer cells, and lead to more mutations that may promote cancer progression. The defective HR pathway offers an excellent condition for PARPis. Epidemiological investigation shows that 50% high-grade serous ovarian cancer (HGSOC) have latent defects in HR repair pathway, which result in one or more germ-line or somatic mutations in BRCA or other DDR-related genes (18). Moreover, 10%–20% breast cancer (mainly triple-negative breast cancer), metastatic prostate cancer, or pancreas cancer have biallelic mutations in HR genes, which leads them also to be sensitive to PARPi. Classically, BRCA1/2 mutated cancer is supposed to respond to PARPis *via* synthetic lethality; that is, PARPis inhibit BER so that SSB converts into DSB subsequently. If cancer cells have underlying HR defects due to BRCA1/2 deficiency, they will be unable to repair DSB and finally die (19). Besides this mechanism, it is also believed that PARPis combine and arrest PARP1 enzyme on chromatin, forming lesions that can only be repaired by HR. Some PARPis like Talazoparib are more effective PARP predators than others (8, 20, 21). Recently, the third mechanism has already been discovered (9, 22). In this case, DSB is excised in S phase as normal. However, cancer cells have to rely on the other DSB repair pathway due to HR defects. It is microhomology-mediated end-joining (MMEJ, also known as Alt-EJ), which depends on PARP1 and DNA polymerase θ (POLQ). In fact, PARP1 is necessary for DSB to recruit POLQ. Therefore, the inhibitors of PARP1 or POLQ block Alt-EJ pathway and kill HR-defective cancer cells. Recent studies show that PARPi-tolerant cancer cells have relatively higher POLQ expression, and they are sensitive to POLQ inhibitor (17).

### Cisplatin

Cisplatin is another type of widely used chemotherapy drugs targeting DDR. Similar to other DDR-based drugs, cisplatin is activated after entering cells. In cytoplasm, the atomic chlorine of cisplatin is replaced by water (23). The aquo complex is a potent electrophile that can react with any nucleophile, including the sulfhydryl of proteins and nitrogen donor atoms of nucleic acid. Cisplatin combines with the N7 reaction center of purine residues, causing DNA damage in cancer cells, inhibiting cell proliferation and finally leading to cell apoptosis. In this process, the 1,2-intrastrand crosslinking between purine bases and cisplatin is the most apparent DNA change (24). It includes 1,2-intrastrand d(GpG) adducts and 1,2-intrastrand d(ApG) adducts, accounting for 90% and 10%, respectively. According to the report, 1,3-intrastrand d(GpXpG) adducts and others (e.g., inter-strand and non-functional adducts) have contributions to the toxicity of cisplatin (25). Despite that it has been observed that cisplatin impairs cancer cells through many aspects, many published results still support that DNA is the key target of cisplatin toxicity.

### DDR Combination Treatment

Besides single-drug treatment, the combination therapy of multiple drugs is a remarkable direction in the future. Many types of tumors like GBM is treated by radiotherapy and TMZ for now. Despite of this, the prognosis of patients is still unsatisfactory, indicating that more studies on combination therapy are required to improve the therapeutic outcome of cancer especially advanced cancer. In the GBM mouse model, people found that PARPis had synergistic effects with radiotherapy and TMZ, slowing down tumor growth and improving patients’ survival (26). In various types of cancer models, PARPis can play as a good radiosensitizer, which significantly raises the mortality of tumor cells (27). Their effects include inhibiting tumor cell proliferation, reducing the survival rate of clones, slowing tumor growth, and improving the survival rate of mice. This also indicates that the combined application of chemotherapy drugs will play an important role in the prospective cancer therapy.

## Application and Progress of the Combination of DDR Anti-tumor Drugs and Immunotherapy

### Applications and Research Progress of Cancer Immunotherapy

The interaction between tumor and immune system is dynamic during tumor formation and progression. Thus, cancer limitation of the immune system and the immune escape of cancer are also in a dynamic balance. Tumor-infiltrated lymphocytes (TILs) have important functions in the dynamic control of tumor. A great number of studies have found that TILs could predict the response of tumors to immunotherapy (28), especially CD8+ T lymphocytes. As the core of anti-tumor immune response, its ability to infiltrate tumors is related to the survival rate of patients (29). Specifically activated CD8+ lymphocytes induce the basic anti-tumor response to cope with the infiltration of tumor antigen. Therefore, TILs are the key to restrict tumor growth. TAA recognized by T lymphocyte induces the specific immune response. Further studies discover that growing tumors contain TILs. They are invalid to eliminate cancer cells *in vivo*, but they can proliferate and function when being removed from the immune-suppressive microenvironment. This is because cancer cells have developed the mechanisms to avoid being recognized and eliminated, including downregulating the components that process and present antigens; recruiting inhibitive immunocytes like regulative T cell, MDSC, and TAM; and producing soluble cytokines involved in immune suppression like transforming growth factor beta (TGF-β) and interleukin (IL)-10. Meanwhile, cancer cells also downregulate the expression like PD-L1.

**Figure 2:**
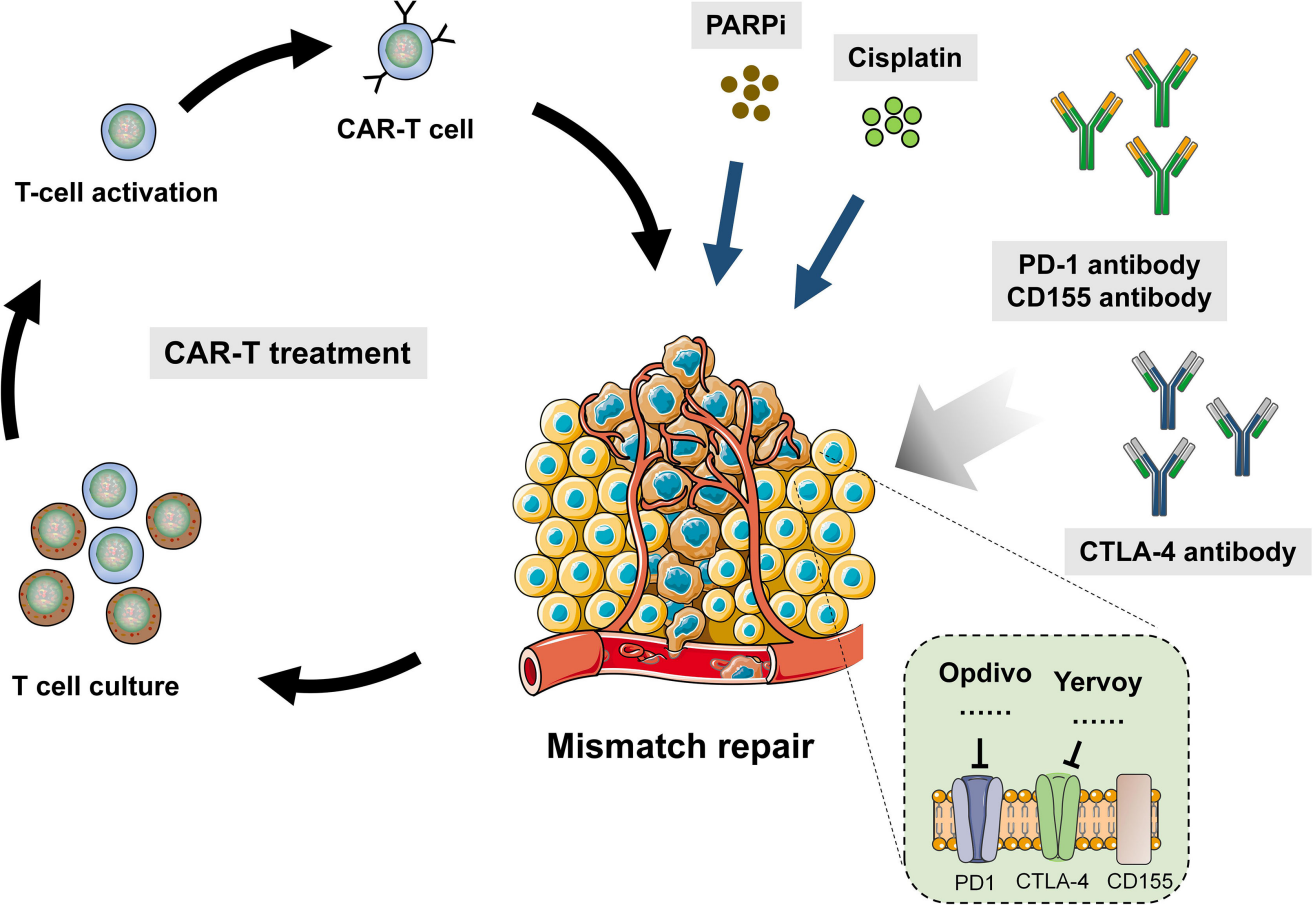
Combination of DDR drug and immunotherapy in human cancer.

It has achieved great success to use CTLA-4 and PD-1 in cancer immunotherapy. Pierre Goldstein discovered CTLA-4 in 1987. Later on, several groups independently proves that CTLA4 played a role as inhibitive receptor both *in vitro* and in gene knockout mice. These led James Allison to start a pioneering work in 1996, which demonstrated that CTLA-4 blocker could eliminate tumors in mice and provide theoretical basis for the clinical development of CTLA-4 antibody. In 2011, FDA approved CTLA-4 antibody (ipilimumab) to be applied in melanoma, which marked a new era of cancer immunotherapy. At present, people has raised many models to explain the mechanism of CTLA-4 functions. The most simple one is the competition between CD28 and CTLA-4. It is similar to other endogenous inhibitive signaling model. Although CTLA-4-defective mice have overactive CD4+ T cells exhibiting pathogenetic clinical phenotypes, chimera can still prevent diseases and normalize the phenotype of defective cells by the part of cells expressing CTLA-4. Consistent with cellular structure, there is another molecular mechanism to capture CD80 and CD86 physically and then remove them from antigen-presenting cells (APC), which is called trans-endocytosis (30). In this procedure, T cells recognize certain peptides and present them to APC. In this way, CD80 and CD86 expression in APC is regulated. Despite that the molecular mechanism by which endocytosis controls gene transcription is unknown (31), there are other receptor–ligand pairs employing this pathway besides CTLA-4. It was found that the Notch–Delta pathway was also one of them. The combination with Delta (ligand) removes Notch (receptor) from neighbor cells (32) (**Figure 2**).

### PD-1 and PD-L1 Drug Treatment

The clinical development of PD-1 inhibitor owes to a series of discovery in basic science. It was first cloned by Tasuku Honjo in 1992, and its ligand (PD-L1) was discovered by two groups led by Lieping Chen and Gordon Freeman independently approximately 10 years later (32). Chen further proved that many human cancers upregulate dPD-L1, and the blocking by its antibody resulted in tumor regression. These findings lay the root for the successful clinical results that PD-1 blocking treats advanced solid tumors. PD-1 is a member of B7/CD28 costimulatory receptor family. It combines the ligand including PD-L1 and PD-L2 to regulate the activation of T cells (33). Like CTLA-4 signaling, the binding of PD-1 and its ligands inhibits T cells to proliferate; reduces the production of interferon gamma (IFN-γ), tumor necrosis factor alpha (TNF-α), and IL-2; and also lowers the survival rate of T cells. If the presentation and recognition of TCR and the combination of PD-1 happens simultaneously in T cells, PD-1 signaling will prevent critical TCR signaling intermediate from phosphorylation. Thus, the early TCR signaling is suspended, and T-cell activation decreases. In normal cells, the main function of PD-1 is making the CD4+ T cells that experience high-level stimulation to be exhausted (34). It also happens in patients with chronic infection of malignant tumors. The characteristic is T-cell dysfunction, resulting in inadequate control for them. As the target of PD-L1 inhibitor, the ligand PD-L1 expression is a natural potential biomarker. However, in the present studies, there are contradictory conclusions. According to the expression of PD-L1 in melanoma, NSCLC, and urogenital cancer and the sensitivity of their response to PD-L1 inhibitor, the recurrent ratio of PD-L1 positive and negative cancer is 34.1% and 19.9%, respectively. Cancer types and certain CPI make some differences, while the lack of standardized test and the heterogeneity of tumor itself may also be one of the reasons that cause these inconsistent results. Collectively, the real value of PD-1 as a biomarker in prediction is still uncertain. Other features of tumor microenvironment like the infiltration of effector immunocytes and the expression of inflammatory genes can also suggest stronger activity. For now, people are studying the functions of some molecules in inflammatory reactions and looking for potential biomarkers in CPI treatment.

CAR technique was first reported by Zelig Eshhar and colleagues in 1993. They used chimeric genes coding single-strand antibody to transduct T cell. This single-strand antibody connects trans-membrane region and the intracellular domain coding T-cell receptor adaptor. It proved that CAR-T enables T cells to redirect to the cells expressing antibody-related antigen (35). The subsequent studies found that CD19 CAR-mediated human peripheral blood T lymphocytes can eradicate the lymphoma and leukemia in immune-compromised mice. In 2010, a case report revealed that the therapeutic effect of CD19 CAR-T in lymphoma patients was very satisfactory. From then, CAR-T has shown impressive results in recurrent or refractory B-cell malignant tumors such as acute and chronic lymphocytic leukemia. CAR-T cells targeting solid tumors have also been tested but has not shown an ideal result yet. According to the costimulatory molecules (CM), CAR-T cells can be classified into four generations (36). However, due to lacking assistance of CM, infused CAR-T cells are poor at proliferation, resulting in unsatisfying results. The second generation of CAR-T has CM like CD28, CD27, and 41BB (CDB7). They can add OX40 (CD134) or inducible CM to T cells in order to overcome the problems of the first generation. Therefore, the second generation has significantly improved in killing cancer cells compared to the first one. As for the third generation, another CM such as CD28, 4-1BB, or CD3 is added to T cells so that they contain two CMs resulting in the further improvement of T-cell proliferation activity, cytotoxicity, and survival rate. The fourth generation is called TRUCKs. Compared to the structures of previous CAR-T, it has additional proinflammatory cytokines such as IL-12 and CM ligand (4-1 BBL and CD40L), which enable CAR-T cells to be released to kill cancer cells. Moreover, the fourth generation saves the pretreatment like routine or high-dose chemotherapy, which raises patients’ quality of life (37).

### Combination of DDR Anti-Tumor Drugs and Immunotherapy

As the studies go further, people gradually found that the mechanisms of DNA damage and immunology are connected in some aspects. Therefore, they came up with combined therapy to treat the cancer that is insensitive to DNA repair-related drugs or immunotherapy. As immunology, especially tumor immunology, develops in recent years, it has been a hot spot to combine immunotherapy and DNA repair, which is proved in defective cancer. Among the drugs, immune checkpoint inhibitors exhibit high activity. CPI is a type of drug that targets the negative inhibitive receptors on T lymphocytes in the host, such as CTLA-4 and PD-1. These receptors are often abducted by cancer in case of effective anti-tumor response (38). Monoclonal antibodies that can block these checkpoints have been developed and show impactful ability to induce deep and enduring response in some late-stage refractory cancer. Consequently, it is approved in many subtypes of cancer including melanoma, NSCLC, HNSCC, RCC, UC, and Hodgkin’s lymphoma. Despite of the excellent performances in these cancers, it seems that only a few patients can benefit from this in clinical trials. It is still vital to develop reliable biomarkers to predict patients’ response and help in selection; these findings also lay foundation for studying the other sources of genome instability, which may also be used as biomarkers for selecting patients who should accept immunotherapy.

More and more evidence shows that HR-defective cancer cells have stronger immunogenicity and have potential effects on CPI. In HGSOC, BRCA1/2-mutated cells have higher predictive new antigen load and more TILs infiltration. In addition, the expression of PD-1/PD-L1 is enhanced (39). A similar increased TIL is also observed in DNA repair-defective breast cancer. In PDA, the transcriptome analysis suggests that the DSB repair-defective subtype is associated with anti-tumor immunological genetic markers (40). A recent large-scale study using the next-generation sequencing to analyze various types of cancer shows that at least 25% HR genes have defects. The TMB of HRD group is significantly higher than that of non-HRD one. People also found that BRCA/Fanconi anemia pathway defective breast cancer cells also have increased cytoplasmic DNA associated with cGAS/STING/TBK1/IRF3 pathway (41, 42).

By affecting TILs, especially the functions and composition of CTLs, PARPi may have immune-suppressive functions or improve anti-tumor response. Anti-PD-1/PD-L1 may deteriorate the former and have synergistic effect with the latter. If PARPis enhance immune response, anti CTLA-4 can coordinate with it and strengthen the effect (43). Higuchi et al. revealed that the binding of anti PD-L1 and PARPis did not induce anti-tumor response. However, the latest studies provide a new perspective on the combination of anti PD-1/PD-L1 and PARPi (Olaparib). This therapy was tested on triple-negative breast cancer (TNBC) both *in vivo* and *in vitro*. The histological results showed that protein formylation in human breast cancer is negatively related to the expression of PD-L1 (20, 44). When inoculated to homogenic mice model, PARP upregulated PD-L1 on the surface of EMT6 cancer cells (TNBC cell line) both *in vitro* and *in vivo*, which was mediated by inactive GSK3β pathway and induced TIL to decrease. Therefore, PARPi suppresses immunity by the decrease in TILs. Compared to PARPi or anti PD-L1 alone, anti PD-L1 may reverse the inhibitive functions of TIL and enhance anti-tumor response when combined with PARPis. These data collectively support further studies on the combination of PARPis and anti PD-L1/PD-1. A recent clinical trial in Stage I applied anti-PD-1 durvalumab and olaparib into patients with breast cancer, establishing good tolerance and achieving improvement in therapeutic effects (44). At present, two combinations have been used in the study of breast cancer therapy, Veliparib and anti-CTLA-4, and Olaparib and anti-PD-L1. Noticeably, immunotherapy is applied in both situations. In future studies, we need to choose different kinds of PARPis according to the cancer types and need more studies to confirm the influences of PARPis on anti-tumor response.

In the *in vitro* experiments, IFNγ, TNFα, and PARPis (Veliparib) can coordinate to inhibit the proliferation of BRCA-deficient cancer cell line. In the mice model inoculated in this cancer cell line, the synergistic effects of anti CTLA-4 and PARPis have better anti-tumor results than any single drug, and the mice in this groups have the highest survival rate. This response is mediated by the infiltration of intraperitoneal CD8+ T cells that produce IFNγ. As the response to anti CTLA-4 and PARPis, the increase in local IFNγ is competent to prohibit tumor growth, while this drug combination does not have similar roles to BRCA1-sufficient ovarian cancer. For now, there is no conclusion if it functions in the immunotherapy in human. Therefore, more studies are required to confirm the roles of PARPis on anti-tumor response.

Combining PARPi and immunotherapy has been the latest progress. In contrast, the synergistic effects of radiotherapy and immunotherapy have been fully vindicated (45). Many studies based on animal models have shown that radiation has anti-tumor immunological effects, mainly by regulating CD8+ T lymphocytes. Besides, ionizing radiation induces proinflammatory lesions and fibration, which is partially mediated by regulating cytokines. A similar process has been observed in cancer, with the infiltration of leukocytes (7, 46). Ionizing radiation activates CTl by a variety of mechanisms, including inducing TAA production and killing tumor-specific T cells. In addition, exposure in ionizing radiation upregulates major histocompatibility complex 1 (MHC-1) expression in cancer cells so that the TAA presenting of CD8+ lymphocytes is enhanced. Ionizing radiation also regulates damage-associated molecular patterns (DAMPs) release in the tumor bed, which then activate macrophages and DC. Finally, CTL production is raised. Ionizing radiation can also upregulate MHC expression to raise the production of TAA. It can improve immuno-response and coordinate with immunotherapy. Since TTA might be caused by cancer mutations, the tumors with higher mutations may have better response to the combination of ionizing radiation and immunotherapy. Not only IR but also cytotoxic effector molecules targeting DNA repair such as PARPis can enhance the mutation load in tumors. DNA repair is already defective. Therefore, PARPis and ionizing radiation may improve therapeutic effects as immune checkpoint inhibitors.

Since the coupling of radiotherapy and PARPis has become a used treatment, its influences on anti-tumor immune response are also required to be tested. Studies have shown that PARPis and radiation can upregulate the expression and secretion of chemokines, such as CCL2 and CCL5 (PARPis, CXCL-16, and CXCL-10), and facilitate tumor infiltrating by CTL. The effects of this group is undetermined. PARPis and ionizing radiation can upregulate PD-L1 on the surface of cancer cells to suppress immunity (45). Thereby, we should investigate the changes in immunological molecular spectra in cancer cells after the induction of radiation plus PARPis and TIL (especially CTL), so that we can gain insight into the underlying mechanisms (6, 17). Animal models are needed to be developed in order to study the effects of triple therapy incorporating PARPis, radiation, and immune checkpoint inhibitors or T cell therapy, and confirm if they can improve anti-tumor response. In this situation, it is quite meaningful to compare the effects of heavily charged particles and photon radiation on anti-tumor immune response and to prove that the combination of radiosensitizer and PARPis can reduce radiotoxicity.

By far, dMMR has still been the only proven genome biomarker that responds to CPI. A large portion of cancer has profound and lasting response. The abnormal high TMB may be the basis of the immunogenecity and instructs a new direction for exploring other genomic biomarkers especially other cancer-related DNA repair pathways defects (47). However, how DNA damage and repair affect the immunogenicity of these cancer seems to be versatile and complex. Particularly, how the different features of DNA damage affect the immunogenicity is largely unknown. In a study in which 60 patients with UC accept PD-1 or PD-L1 inhibitor treatment, the targeting exon sequencing shows that the existence of DDR changes is associated with 67.9% recurrence. Different reaction pathways make different influences. The effects are maximum in the cancer with POLE or NER mutation. Prospective study is required to confirm the contribution of defects in certain pathway in the possibility of CPI response.

Now, there are some ongoing clinical trials on DDR-related drugs with immunotherapy (**Table 2**). However, currently, both DDR and immunotherapeutic drugs have their limitations and resistance reported. Resistance to DDR drugs has now been widely reported, while immunotherapy, especially PD-1 inhibitors, has not been very effective in some solid tumors. For example, although some studies have shown that PD-1 and chemotherapeutic agents such as cisplatin can improve the survival time of some patients with nasopharyngeal carcinoma, the expression of PDL1 in most patients with nasopharyngeal carcinoma is low. Especially for recurrent nasopharyngeal carcinoma, PD-1 inhibitor does not show a good effect respond. This also needs to be further explored in future studies.

**Table 2:** Ongoing clinical trials on DDR-related drugs with immunotherapy.

Research title	Tumor type	Drugs	Locations
Camrelizumab Combined With Apatinib, Etoposide and Cisplatin Treat Small-cell Lung Cancer.	Lung Neoplasm	Camrelizumab; Apatinib; Etoposide; Cisplatin	The 900th Hospital of Joint Logistic Support Force Fuzhou, Fujian, China
	Small Cell Lung Cancer		
Anlotinib in Combination With PD1 With Gemcitabine Plus(+)Cisplatin for Unresectable or Metastatic Biliary Tract Cancer	Biliary Tract Cancer	PD1 inhibitor; Cisplatin	Zhejiang Cancer Hospital Hangzhou, Zhejiang, China
Concurrent Immunotherapy With Postoperative Radiotherapy in Intermediate/High Risk HNSCC Patients Unfit for Cisplatin: The IMPORT Study (IMPORT)	Head and Neck Squamous Cell Carcinoma	PD1 inhibitor; Cisplatin	Guopei Zhu Shanghai, China
Neoadjuvant Anti-PD-1 and TP Versus TPF on Pathological Response in OSCC	Oral Squamous Cell Carcinoma	Toripalimab; Albumin paclitaxel; Cisplatin	Ninth People’s Hospital, Shanghai Jiao Tong University School of Medicine Shanghai, Shanghai, China
A Clinical Trial Comparing HLX10 With Placebo Combined With Chemotherapy (Cisplatin + 5-fu) in the First-line Treatment of Locally Advanced/Metastatic Esophageal Squamous Cell Carcinoma (ESCC)	Esophageal Squamous Cell Carcinoma	HLX10; Cisplatin	Ethics Committee of cancer hospital, Chinese academy of medical sciences, Beijing, Beijing, China et al.
Camrelizumab Combined With Chemotherapy for Recurrent or Advanced Cervical Neuroendocrine Carcinomas	Cervical Neuroendocrine Carcinoma	Camrelizumab; Cisplatin	Lei Li Beijing, Beijing, China
Efficacy and Safety of BCD-100 (Anti-PD-1) in Combination With Platinum-Based Chemotherapy as First Line Treatment in Patients With Advanced Non-Squamous NSCLC	Non-Squamous Non-Small Cell Neoplasm of Lung	Pemetrexed; Cisplatin (or carboplatin)	Regional Hospital Liberec
			Liberec, Czechia University Hospital Olomouc, Czechia et al.
Toripalimab Combined With Gemcitabine and Cisplatin Treating Resectable Locally Advanced HNSCC	Locally Advanced Head and Neck Squamous Cell Carcinoma	PD-1 inhibitor; Gemcitabine; Cisplatin	Fifth Affilliated Hospital of Sun Yat-sen University Zhuhai, Guangdong, China
Perioperative Pembrolizumab (MK-3475) Plus Neoadjuvant Chemotherapy Versus Perioperative Placebo Plus Neoadjuvant Chemotherapy for Cisplatin-eligible Muscle-invasive Bladder Cancer (MIBC) (MK-3475-866/KEYNOTE-866)	Urinary Bladder Cancer, Muscle-invasive	Pembrolizumab; Gemcitabine; Cisplatin	Scripps MD Anderson, California, United States et al.
Clinical Study of Camrelizumab in Combination With Neoadjuvant Chemotherapy for Operable Locally Advanced Head and Neck Squamous Cell Carcinoma	Head and Neck Cancer	PD-1 inhibitor; Albumin Paclitaxel; Cisplatin	Hunan cancer Hospital Changsha, Hunan, China
	Squamous Cell Carcinoma		
Efficacy and Safety of Pembrolizumab Plus Investigational Agents in Combination With Chemotherapy as First-Line Treatment in Extensive-Stage Small Cell Lung Cancer (ES-SCLC) (MK-3475-B99/KEYNOTE-B99)	Small Cell Lung Cancer	Pembrolizumab; MK-4830; MK-5890	Banner MD Anderson Cancer Center Gilbert, Arizona, United States et al.
Placebo-controlled, Study of Concurrent Chemoradiation Therapy With Pembrolizumab Followed by Pembrolizumab and Olaparib in Newly Diagnosed Treatment-Naïve Limited-Stage Small Cell Lung Cancer (LS-SCLC) (MK 7339-013/KEYLYNK-013)	Small Cell Lung Cancer	Pembrolizumab; Olaparib	Ironwood Cancer & Research Centers et al.
Study of Pembrolizumab With Concurrent Chemoradiation Therapy Followed by Pembrolizumab With or Without Olaparib in Stage III Non-Small Cell Lung Cancer (NSCLC) (MK-7339-012/KEYLYNK-012)	Lung Neoplasms Carcinoma, Non-Small-Cell Lung	Pembrolizumab; Olaparib	University of South Alabama, Mitchell Cancer Institute, Alabama, United States et al.
Phase II Umbrella Study of Novel Anti-cancer Agents in Patients With NSCLC Who Progressed on an Anti-PD-1/PD-L1 Containing Therapy	Non-Small Cell Lung Cancer	Durvalumab; AZD9150; AZD6738	Research Site Duarte, California, United States et al.
Phase II Study of Olaparib and Pembrolizumab in Advanced Melanoma With Homologous Recombination (HR) Mutation	Metastatic Melanoma	Olaparib; Pembrolizumab	California Pacific Medical Center Research Institute, San Francisco, California, United States
Paclitaxel, Pembrolizumab and Olaparib in Previously Treated Advanced Gastric Adenocarcinoma	Advanced Gastric Adenocarcinoma	Paclitaxel; Olaparib; Pembrolizumab	Sidney Kimmel Comprehensive Cancer Center Baltimore, Maryland, United States

## Conclusions and Future Perspectives

In the past decades, we have made great progress on understanding how cancer cells escape from the surveillance of immune system, which offers us new approaches based on how to prevent immune escape and eliminate cancer cells. The immune system has vital functions in the progression and restriction of cancer. The immunological surveillance of cancer includes three stages, namely, elimination, balance, and escape (48, 49). In this process, IFNγ and lymphocytes inhibit primary tumor progression. In the stage of elimination, the immuno-response can induce effective exogenous tumor-suppressing system, while the bad side is that it also causes an immunological selection of cancer cells that are more capable to survive in the immune-competent host. Next, the chosen tumor cells enter the stage of immune escape. Between these two stages, it is called immune balancing stage, in which tumor does not grow or grow slowly under the pressure of the immune system (50). Both innate and adaptive immunity particulate in countering cancer. Besides, cytokine IFNγ has pleiotropic effects, especially in the activation of NK cells and CTL. IFNγ signaling upregulates the expression of MHC I and MHC II and raises the expression of TAA in lymphocytes. IFNγ also induces specific TAA-activated CD8+ lymphocytes, thereby initiating CTL-mediated anti-tumor response. CTL infiltration enhances the lethal effect of immune system to cancer cells. A body of evidence has shown that CTL infiltration is related to the good prognosis of many tumors (51–53).

So far, people have found that MDSC plays an important role in tumorigenesis and progression as well besides TILs. Studies show MDSC can directly support the growth and metastasis of tumor besides suppressing the immune system. Similar to TILs, MDSC can also be considered as a factor for prognosis of cancer immunotherapy and the target of drugs according to the clinical data of patients. In sum, tumor-related myeloid including MDSC and TAM reprogram to create an immune-suppressing environment in tumorigenesis. Meanwhile, it also promotes tumor stem, angiogenesis, the transformation from epithelium to mesenchyme and metastasis, which directly drive the progression of tumors. As an important kind of RNA, noncoding RNA also may play an important role in tumor treatment. Non-coding RNAs have been found to play important functions in tumor immunity and DNA damage repair in a variety of tumors, such as lncRNA and circRNA, which suggest that they may serve as important targets for combination drug delivery.

DDR drugs cause biological behaviors such as apoptosis of tumor cells through DNA damage, thus leading to the death of tumor cells. In contrast, immunosuppressants, especially immune checkpoint inhibitors represented by PD-1, promote the killing of tumor cells by immune cells *via* attenuating the immune escape of tumor cells. Recent studies also have found that DDR drugs lead to changes in tumor microenvironment, which mechanistically explains why the combination of DDR drugs and immunotherapy may have better effects on the treatment of tumors. The common pathway between DDR and immunotherapy has also been one of the main focuses of research in recent years, and studies have identified molecules represented by cGAS, which play important functions in both DNA damage repair and immunity. The roles of cGAS in immunity and DNA repair is noteworthy. Previous studies reported the existence of cytoplasmic DNA damage in late S phase. In addition, there has been evidence proving that cells can actively export DNA segment from the nucleus, which possibly contributes to the prevention of incorporating mistakes into DNA. Reversely, when efficient DNA repair is lacking, cytoplasmic DNA will trigger cGAS-mediated innate immuno-response (54, 55). Studies have shown that the downregulation of cGAS and STING successfully reverses the effects of combining DNA repair-related anti-tumor drugs and PD-L1 in prohibiting tumor growth. Therefore, cGAS can also be a molecule worth further investigation. However, although the combination of immunotherapy and DDR drugs has achieved good results in some tumors, current clinical studies have shown that most solid tumors are not sensitive to immunotherapy or immunotherapy plus chemotherapy, which requires further investigation of the mechanisms and development of new immunotherapy drugs in future studies.

## Author Contributions

YW, MD and ZP collected the related paper and finished the manuscript and figures. YH, WX, HZ and RF gave constructive guidance and made critical revisions. WJ participated in the design of this review. All authors read and approved the final manuscript.

## Funding

This research was funded by the National Natural Science Foundation of China (81770985), Xiangya Hospital Funds for Young Scholar (2020Q13), Hunan Postdoctoral Program for Innovative Talent (2021RC2017), and Natural Science Foundation of Hunan Province (2021JJ41027). The funders had no role in study design, data collection and analysis, decision to publish, or preparation of the manuscript.

## Conflict of Interest

The authors declare that the research was conducted in the absence of any commercial or financial relationships that could be construed as a potential conflict of interest.

## Publisher’s Note

All claims expressed in this article are solely those of the authors and do not necessarily represent those of their affiliated organizations, or those of the publisher, the editors and the reviewers. Any product that may be evaluated in this article, or claim that may be made by its manufacturer, is not guaranteed or endorsed by the publisher.

## Abbreviations

MMR, mismatch repair; HR, homologous recombination; DDR, DNA damage and repair; PD-1, programmed cell death protein 1; MGMT, O-6-methylguanine-DNA methyltransferase; NSCLC, non-small cell lung cancer; HNSCC, head and neck squamous cell carcinoma; RCC, renal cell carcinoma; UC, urothelial carcinoma; PARP, poly-ADP-ribose polymerase; SSB, single-strand break; DSB, double-strand break; PARP, poly-ADP-ribose polymerase; TIL, tumor-infiltrating lymphocyte; PD-L1, programmed cell death-ligand 1; cGAS, cyclic GMP-AMP synthase; circRNA, circular RNA; lncRNA, long noncoding RNA.
